# Patient treatment engagement scale (PTES) for inflammatory bowel disease: exploring background factors

**DOI:** 10.1186/s13104-026-07664-2

**Published:** 2026-09-21

**Authors:** Hisao Toyoshima, Chiharu Yashima, Tomohide Akase, Masayoshi Koinuma

**Affiliations:** 1 Pharmacy Management Institute, Graduate School of Business, Japan University of Economics, 25-17 Sakuragaokacho, Shibuya-Ku, Tokyo, Japan; 2 Faculty of Pharmaceutical Sciences, Teikyo Heisei University, Tokyo, Japan; 3 Graduate School of Business , Japan University of Economics, Tokyo, Japan

**Keywords:** Patient treatment engagement scale (PTES), Inflammatory bowel disease (IBD), Ulcerative colitis (UC), Crohn’s disease (CD)

## Abstract

**Background:**

The Patient Treatment Engagement Scale (PTES) is a simplified tool for assessing patient involvement in treatment decision-making, which is an important element of patient-centered care. Shared Decision Making (SDM) is the standard measure in this field; however, it is time-consuming and often difficult for non-physician healthcare professionals to apply in routine practice. Therefore, in this study we adopted PTES as a more pragmatic alternative and exploratively analyzed factors associated with PTES scores among patients with inflammatory bowel disease (IBD).

**Methods:**

Individual patient data were collected via a web-based questionnaire. Items potentially associated with PTES, based on previous studies, were screened, and multicollinearity was assessed using Kendall’s τ. A multiple regression model was constructed using the ordinary least squares method, and standardized β values and variance inflation factor (VIF) values were calculated. Subgroup analyses were performed separately for ulcerative colitis (UC) and Crohn’s disease (CD), and cross-validation was conducted.

**Results:**

The main factors associated with PTES scores were the number of treatment options (β = 0.191), presence of complications (β = 0.128), trust in the attending physician (β = 0.119), number of trusted healthcare professionals (β = 0.099), and availability of supportive communities (β = 0.068). The number of treatment options had a particularly strong influence on UC patients, whereas the number of trusted healthcare professionals had a greater impact on CD patients.

**Conclusions:**

Providing multiple treatment options and building trust with healthcare providers are crucial factors associated with higher PTES scores, reflecting greater patient engagement in treatment decision-making.

**Supplementary Information:**

The online version contains supplementary material available at https://doi.org/10.1186/s13104-026-07664-2.

## Background

Shared Decision Making (SDM) is a process where patients and healthcare providers share information to make treatment decisions that respect patient values [1]. It is between paternalistic decision-making, where doctors decide alone, and informed consent, where patients decide. SDM is important for treatment adherence and improving quality of life (QOL) in chronic diseases or conditions with multiple treatment options.

Many guidelines recommend SDM. It is vital to provide sufficient information and understand patients’ goals. Long-term treatment plans should involve patients and, with consent, their families. After agreement, treatment plans should be made to prevent relapse, manage symptoms, and improve QOL, with therapy tailored to the patient’s needs [2]. UC patients often struggle with inadequate educational information and social support during complex treatment decisions [3].

However, research on factors affecting SDM engagement in inflammatory bowel disease (IBD) is limited. Studies show that age, education, and disease knowledge influence SDM participation. Younger, more educated patients engage more actively, while older or less educated patients may need more support. More research on specific factors is needed.

Even the 9-item SDM-Q-9 [4] can burden routine multidisciplinary use—hence our single-item proxy. In particular, non-physician healthcare professionals, such as pharmacists, also play an important role in supporting patients’ treatment engagement. Therefore, we concluded that a simple tool that closely resembles SDM is necessary to assess the degree of patient involvement in treatment decisions and function as a practical aid in clinical settings. Based on this rationale, we operationalized a single-item Patient Treatment Engagement Scale (PTES) as a pragmatic proxy for engagement in treatment decision-making with the primary aim of identifying disease-specific differences between ulcerative colitis (UC) and Crohn’s disease (CD) as key factors that enhance PTES.

## Methods

This study is a secondary analysis of previously collected data [5], focusing on factors influencing Shared Decision Making (SDM). An anonymous online survey was conducted in March 2022 targeting 5000 inflammatory bowel disease (IBD) patients, aged 17 or older, with ulcerative colitis (UC), Crohn’s disease (CD), or indeterminate colitis. Informed consent was obtained from all participants.The data from 557 eligible respondents were analyzed.

The survey was commissioned to a research company (Macromill, Inc., Tokyo, Japan), which has approximately 1.3 million registered participants and has obtained the Privacy Mark certification, ensuring appropriate data protection. The effective response rate was 100% with a continuation rate of 99.8%. While these figures are comparable to previous web-based patient-reported outcome surveys, we fully acknowledge the inherent risk of sampling bias in internet-based surveys. In particular, patients with higher digital literacy may be disproportionately represented.

## Measures

Participants were asked about their involvement in treatment decisions (on a five-point scale), the number of treatment options presented, and the medical staff they trust. They also reported complications, income, employment, education, and family status. The MHC-SF index was used to assess psychological and physical health, with questions on motor function, nutritional status, and oral function.

**Fig. 1:**
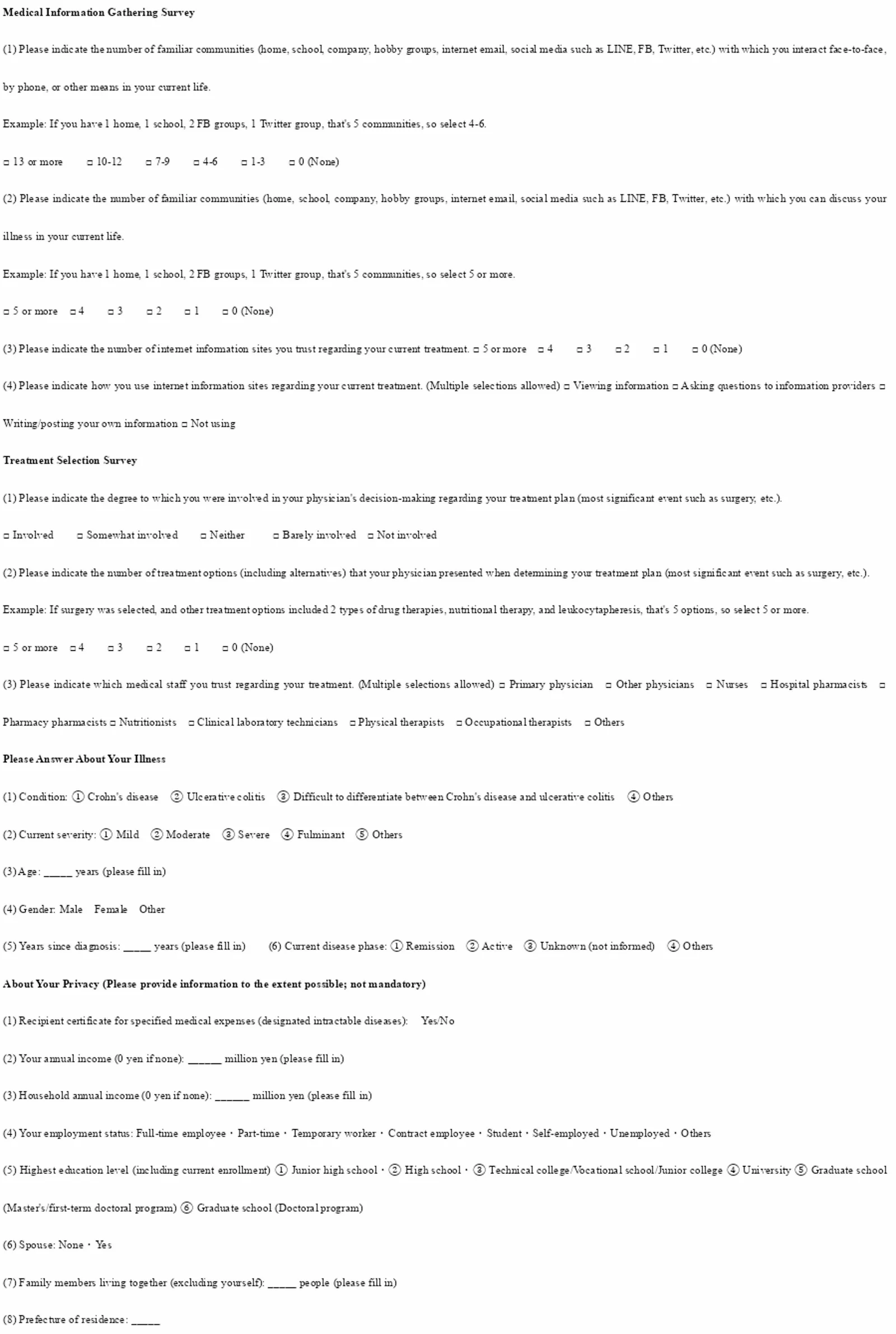
Questionnaire

The questionnaire was a self-administered survey that included items on medical information gathering, treatment selection, medical history, and privacy-related questions (Fig. 1). The response format consisted of multiple-choice or free-response items, and the survey was conducted online with the consent of the participants. (The complete questionnaire is available in the Supplementary Materials.)

### Statistical analysis

Data were analyzed using JMP Pro 16.2 with statistical significance set at 5%. Strongly correlated variables (|τ|> 0.5) were screened for multicollinearity, and only one from each pair was selected [5].

### Explanatory model creation

An explanatory model was created using the least squares method, with patient involvement as the dependent variable. Standardized β values and Variance Inflation Factors (VIF) were calculated. Local data filters were used to compare UC and CD patients [5].

### Model validation

Cross-validation was performed by splitting the data into training (75%) and validation (25%) sets. Explanatory models were created for each set, and β values and VIF were used to assess result stability [6].

### Ethical approval and consent

Ethical approval was obtained from Japan University of Economics (Approval number: 2022–0328-02). Data were anonymized by Macromill, Inc., and participants were informed about data protection at the start of the survey.

## Results

Of the 557 patients analyzed, 108 had Crohn’s disease (CD), 440 had ulcerative colitis (UC), and 9 had indeterminate colitis. The 9 patients with indeterminate colitis were excluded.

The age range was 17–79 years, with a mean age of 50. The mean age for UC patients was 51 years, while for CD patients, it was 47 years.

There were 360 male and 188 female participants. These characteristics are summarized in Table 1.

**Table 1:** Participant characteristics

					95％CI	
		Number	M	±SD	Lower limit	Upper limit
Final education	Graduate school (doctorate)	6	30.33	20.66	8.66	52.01
	Graduate school (Master Master's degree)	22	27.05	18.86	18.68	35.41
	University	255	25.40	18.16	23.16	27.64
	Technical college/vocational school/junior college	119	23.72	16.65	20.70	26.74
	High school	145	19.18	17.10	16.37	21.99
	Secondary school	10	19.00	18.93	5.46	32.54
Gender	Male	366	23.70	18.24	21.83	25.58
	Female	191	22.89	16.90	20.48	25.30
Married	Married	215	18.32	16.25	16.13	20.50
	Never married (including bereavement/separation)	342	26.63	17.97	24.72	28.55
Children	Yes	255	20.05	16.20	18.06	22.05
	No	302	26.27	18.57	24.17	28.37
Job	Freelance	14	29.50	20.05	17.92	41.08
	Student	6	27.83	11.44	15.82	39.84
	Manager/officer	17	27.71	16.59	19.17	36.24
	Others	5	26.40	15.66	6.95	45.85
	Company employee (clerical)	118	25.80	19.41	22.26	29.34
	Company employee (other)	64	24.02	17.39	19.67	28.36
	Company employee (technical)	59	23.83	18.30	19.06	28.60
	Self-employed	32	23.53	16.78	17.48	29.58
	Housewife (househusband)	50	22.88	17.55	17.89	27.87
	Public servant	29	22.62	16.42	16.37	28.87
	Part-time job	69	22.32	17.87	18.03	26.61
	Unemployed	94	18.98	16.45	15.61	22.35
Receiver's card for specific medical expenses (designated intractable disease)	Yes	352	22.67	17.59	20.83	24.52
	No	205	24.71	18.08	22.22	27.20

					95％CI	
		Number	M	±SD	Lower limit	Upper limit
Disease	Crohn's disease	108	21.16	17.53	17.81	24.50
	ulcerative colitis	440	24.04	17.84	22.36	25.71
	IBD unclassified	9	20.67	17.10	7.52	33.81
Seriousness	Mild	359	24.06	17.52	22.24	25.87
	Moderate	143	23.22	17.01	20.41	26.03
	Severe	22	20.41	21.03	11.08	29.73
	Fulminant（most severe of the severely ill）	7	13.43	15.09	− 0.52	27.38
	Don't know/not communicated	21	17.95	21.03	8.38	27.52
	Others	5	34.00	27.72	− 0.42	68.42
Stage	Remission	438	24.01	17.97	22.32	25.70
	Active	86	22.15	17.49	18.40	25.90
	Don't know/not communicated	25	17.44	15.16	11.18	23.70
	Others	8	23.63	16.88	9.52	37.73
Disease complication	Gastrointestinal complications (anal disease, small bowel stricture, etc.)	88	23.65	17.54	19.93	27.36
	Extra-intestinal complications (arthralgia, skin diseases, etc.)	39	26.56	19.42	20.27	32.86
	Cancer	22	30.82	20.92	21.54	40.09
	Mental illness (manic-depression, depression, mania, etc.)	50	19.30	17.31	14.38	24.22
	Sleep disorders	53	21.47	17.69	16.60	26.35
	Reduced musculoskeletal function (orthopaedic and other diseases)	25	20.16	19.64	12.05	28.27
	Undernutrition	35	23.23	19.09	16.67	29.79
	Poor oral function (dental and other diseases)	22	20.18	17.40	12.47	27.89
	Others	14	28.57	23.48	15.01	42.13

M: Mean, SD: Standard deviation, CI: Confidence interval

Socio-demographic background, clinical characteristics MHC-SF-J [mean, standard deviation, lower and upper limits of total score 70] [5]

### Screening and multicollinearity of factors considered

Screening was conducted using the level of involvement of IBD patients themselves in treatment decision-making as the objective variable, resulting in the eight factors listed in Table 2 that were considered to be influential. In the overall IBD screening, variables such as disease severity, intestinal and extraintestinal complications, disease duration, and treatment history were not found to be statistically associated.

**Table 2:** Screening results for patients' own involvement in treatment decision-making

	Frequency	*p*-value	Logarithmic value	FDR P-value	FDR Logarithmic value	Effect size	Fractional rank	R-squared
Number of treatment options	548	< 0.001	9.50488	< 0.001	7.37134	0.23588	0.00735	0.07
Number of trust professions	548	< 0.001	7.17504	< 0.001	5.34253	0.20337	0.01471	0.052
Presence or absence of complications	548	< 0.001	5.31414	< 0.001	3.65773	0.17282	0.02206	0.0376
Trusted medical profession: nurse	548	< 0.001	4.5544	< 0.001	3.02292	0.15865	0.02941	0.0317
Complications gastrointestinal tract	548	< 0.001	4.11606	0.002082	2.6815	0.14988	0.03676	0.0283
Number of communities where you can discuss your illness	548	< 0.001	3.61101	0.004758	2.32257	0.13913	0.05147	0.0244
Trusted medical profession: pharmacy pharmacist	548	< 0.001	3.25626	0.009423	2.02581	0.13108	0.05882	0.0216
Use of information websites: browsing	180	< 0.001	3.15212	0.010646	1.97283	0.20137	0.06618	0.0626
Trusted medical profession: primary care physician	548	0.00216	2.66592	0.022489	1.64802	0.11658	0.09559	0.0171

Multivariate correlations for these eight items were examined to confirm multicollinearity and are shown in Table 3.

**Table 3:** Kendall's rank correlation coefficients to confirm multicollinearity

Variables	vs. Variables	Kendall's rank correlation coefficient (τ)	*p*(Prob >|τ|)	
Number of trust occupations	Nurses	0.7193	< 0.0001	++++++
Number of trust professions	Pharmacy Chemist	0.5218	< 0.0001	+++++
Trusted medical staff physician	Browse information websites	0.4854	< 0.0001	++++
Number of trust professions	Trusted medical staff Doctor	0.3799	< 0.0001	+++
Pharmacy pharmacists	Nurses	0.3486	< 0.0001	+++
Number of trust professions	Number of treatment options	0.2144	< 0.0001	++
No complications	Browsing information websites	0.2032	0.0066	++
Nurses	Number of treatment options	0.1976	< 0.0001	++
Number of treatment options	*Number of communities (contiguous)	0.187	< 0.0001	++
Number of trust professions	*Number of communities (contiguous)	0.1784	< 0.0001	++
Complications Gastrointestinal tract	Number of treatment options	0.1317	0.0006	+
Nurse	*Number of communities (contiguous)	0.1291	0.0009	+
Nurses	Trusted medical staff physician	0.1278	0.0028	+
Pharmacy pharmacist	*Number of communities (contiguous)	0.1239	0.0014	+
No complications	Trusted medical staff physician	0.0969	0.0235	+
Pharmacy Pharmacist	Browsing information websites	0.0963	0.1978	+
Complications Gastrointestinal tract	Pharmacy pharmacist	0.0835	0.0507	+
Number of trust professions	Information website visits	0.0803	0.2409	+
Pharmacy pharmacist	Number of treatment options	0.0692	0.0725	+
Pharmacy pharmacist	Trusted medical staff doctor	0.0658	0.1239	+
Complicated gastrointestinal tract	Number of trusted professions	0.0651	0.1013	+
Complicated gastrointestinal tract	*Number of communities (contiguous)	0.038	0.3264	
Trusted medical staff Physician	*Number of communities (contiguous)	0.0305	0.4309	
Complicated gastrointestinal tract	Nurses	0.0243	0.5704	
Nurse	Browsing information websites	0.0163	0.8279	
Complicated gastrointestinal tract	Trusted medical staff Physician	0.0156	0.7155	
Trusted medical staff Physician	Number of treatment options	− 0.0013	0.9724	
Complicated gastrointestinal tract	Browsing information websites	− 0.005	0.9471	
No complications	Pharmacy Chemist	− 0.0591	0.1665	–
No complications	*Number of communities (contiguous)	− 0.075	0.0526	–
No complications	Number of trust occupations	− 0.1129	0.0045	–
Number of treatment options	Nurses	− 0.1233	0.0039	–
No complications	Number of treatment options	− 0.2215	< 0.0001	–
Number of treatment options	Browsing information websites	− 0.2346	0.0004	–
Information website browsing	*Number of communities (contiguous)	− 0.243	0.0003	–
No complications	Complications Gastrointestinal tract	− 0.6194	< 0.0001	–

For the strongly correlated combinations shaded in Table 3, where the absolute value of Kendall's rank correlation coefficient (τ) exceeds 0.5, three items were eliminated from the eight items in Table 2: trusted nurse, trusted pharmacy pharmacist, and gastrointestinal complications.

### Explanatory modelling.

Model fitting using the least squares method was carried out for the five items not affected by multicollinearity, as shown in Table 4.

**Table 4:** Degree of influence of patient background factors on positive attitudes towards SDM in the population as a whole, ulcerative colitis, and Crohn’s disease

Term	Estimate	Standard error	t-value	*p*-value (Prob >|t|)	Standard β	VIF
Parameter estimates (validation: overall)						
Section	2.9476882	0.123896	23.79	< 0.0001		
Number of treatment options	0.1904855	0.043625	4.37	< 0.0001	0.19102	1.1819
Complications	0.1814493	0.066048	2.75	0.0062	0.12782	1.33683
Trusted medical staff physician	0.2421841	0.087215	2.78	0.0057	0.11928	1.13947
Number of trust professions	0.0988861	0.045815	2.16	0.0313	0.09856	1.28789
Number of communities	0.0634201	0.040089	1.58	0.1142	0.06753	1.1255
Parameter estimates (validation: ulcerative colitis)						
Section	2.8159663	0.152103	18.51	< 0.0001		
Number of treatment options	0.2567118	0.050358	5.1	< 0.0001	0.244	1.152
Trusted medical staff physician	0.2515915	0.103711	2.43	0.016	0.115	1.125
Complications	0.1530945	0.076530	2	0.046	0.095	1.124
Number of trust professions	0.0941621	0.055255	1.7	0.089	0.085	1.244
Number of communities (contiguous)	0.0726626	0.043504	1.67	0.096	0.078	1.101
Parameter estimates (validation: Crohn's disease)						
Section	3.2961629	0.218405	15.09	< 0.0001		
Number of trust professions	0.1643193	0.082059	2	0.0479	0.22662	1.48591
Complications	0.2013251	0.129571	1.55	0.1233	0.14895	1.06612
Trusted medical staff physician	0.2177703	0.156419	1.39	0.1669	0.13835	1.14559
Number of communities (contiguous)	0.0186414	0.103133	0.18	0.8569	0.01899	1.28099
Number of treatment options	− 0.016208	0.087845	− 0.18	0.854	− 0.01952	1.29843

The condition was used as a confounding factor, and differences between conditions were analyzed using the local data filter function in JMP, as shown in Table 4 for ulcerative colitis and Crohn's disease. 

In the combined analysis for ulcerative colitis and Crohn’s disease, the factors most influencing patient involvement in treatment decisions (highest standardized beta values) were: number of treatment options, having complications, trusted physician, and number of trusted medical staff. These four items had a significant impact. As the VIFs for all were below 5, there was no multicollinearity.

**Table 5:** Influence of patient background factors on the willingness to SDM in a randomly selected 75% of the sample (population as a whole, ulcerative colitis, and Crohn’s disease)

Term	Estimate	Standard error	t-value	*p*-value (Prob >|t|)	Standard β	VIF
Parameter estimates (validation: overall)						
Section	2.93299	0.133976	21.89	<0.0001	0	
Number of treatment options	0.2376088	0.052791	4.5	<0.0001	0.228225	1.1985504
Trusted medical staff physician	0.2670814	0.098933	2.7	0.0072	0.13335	1.1374039
Complications	0.1623699	0.069479	2.34	0.0199	0.114352	1.1161326
Number of trust professions	0.0895849	0.05204	1.72	0.0859	0.092049	1.3328138
Number of communities	0.024040	0.047501	0.51	0.6131	0.024784	1.117926
Parameter estimates (validation: ulcerative colitis)						
Section	2.8205875	0.173967	16.21	<0.0001	0	.
Number of treatment options	0.2901531	0.060775	4.77	<0.0001	0.263433	1.1217473
Trusted medical staff physician	0.2337426	0.122174	1.91	0.0566	0.105595	1.1223397
Complications	0.1018656	0.063937	1.59	0.1121	0.093627	1.2723637
Number of trust professions	0.1339032	0.090297	1.48	0.1391	0.081882	1.1233038
Number of communities (contiguous)	0.0439948	0.051806	0.85	0.3964	0.04608	1.0847966
Parameter estimates (validation: Crohn's disease)						
Section	3.2024053	0.23624	13.56	<0.0001	0	.
Trusted medical staff physician	0.3178476	0.166976	1.9	0.0605	0.207955	1.1286907
Number of trust professions	0.1278686	0.093457	1.37	0.175	0.180444	1.6449065
Complications	0.1946166	0.143427	1.36	0.1786	0.143887	1.0634305
Number of treatment options	0.0940443	0.11619	0.81	0.4207	0.106893	1.6494568
Number of communities (contiguous)	-0.064419	0.127377	-0.51	0.6144	-0.06216	1.428931

For ulcerative colitis patients, the most significant factors influencing their involvement in treatment decisions were the number of treatment options, trusted physician, complications, and the number of trusted professionals. The top three factors had a significant impact.

For Crohn’s disease patients, the main factors influencing involvement were the number of trusted professionals, complications, trusted physician, number of communities, and treatment options. The number of trusted professionals had the greatest influence. Compared to ulcerative colitis patients, trust in healthcare professionals was more important, and the number of communities where patients could discuss their illness was more influential, though not significantly so.

### Validation of the models created

Cross-validation was then carried out on a randomly selected 75% sample, the results of which are shown in Table  5.

In cross-validation, the top three most influential factors were similar to the results for the whole population. This suggests that sample size does not have a significant impact on the results.

The degree of impact by disease in the cross-validation population is shown in Table 5 for both the ulcerative colitis and Crohn's disease patient groups.

## Discussion

This study explored factors influencing inflammatory bowel disease (IBD) patients’ engagement in Patient Treatment Engagement Scale(PTES). Key factors identified included the number of treatment options, complications, trust in the primary physician, number of trusted healthcare professionals, and available supportive communities.

The number of treatment options had the greatest impact on SDM engagement, suggesting that offering multiple treatment options may encourage patient participation. Healthcare providers should present a range of options, explaining their benefits and risks.

Complications also influenced SDM engagement, as patients with complex medical needs were more likely to actively participate in decision-making. Healthcare providers should prioritize supporting patients with complications in the decision-making process.

The differences in subgroup analysis results between ulcerative colitis (UC) and Crohn’s disease (CD) patients are particularly interesting. For UC patients, the number of treatment options, trust in the primary physician, and presence of complications significantly influenced engagement, while for CD patients, the number of trusted healthcare professionals had the most pronounced effect. This difference may reflect the characteristics and treatment approaches of the two diseases. CD often requires more complex and diverse therapeutic interventions, such as management of anal lesions and surgery for intestinal strictures [7]. Consequently, CD treatment tends to involve a wider range of specialists beyond gastroenterologists, including surgeons, radiologists, and nutritionists. This complex treatment environment may explain why the number of trusted healthcare professionals strongly influenced SDM engagement in CD patients.

Conversely, UC has a relatively uniform treatment approach, primarily centered on medical therapy [8]. This may explain why the number of treatment options and trust in the primary physician were more important factors for UC patients. The influence of complications on SDM engagement in UC also suggests that disease severity and complexity may increase patients’ willingness to participate in the decision-making process.

The positive influence of trust in the primary physician on SDM engagement reemphasizes the importance of the doctor-patient relationship. Building trust is foundational for patients to actively participate in the SDM process. Healthcare providers need to improve their communication skills and work on building trust with their patients.

The impact of the number of trusted healthcare professionals on SDM engagement highlights the importance of multidisciplinary collaboration. Patients’ trust in multiple healthcare professionals may foster a sense of comprehensive medical support, potentially leading to more active participation in SDM. Healthcare institutions should promote multidisciplinary collaboration and create an environment where patients can receive support from various medical specialists.

The influence of the number of available supportive communities on SDM engagement underscores the importance of patients’ social support. Having communities where IBD patients can discuss their illness may promote their participation in the decision-making process. Healthcare providers should introduce patient support groups and other community resources to help patients build their social support networks.

These findings suggest the need for disease-specific approaches when promoting SDM for IBD patients [9]. For CD patients, strengthening multidisciplinary collaboration and ensuring support from various specialists may be crucial. For UC patients, focusing on presenting sufficient treatment options and building strong trust relationships with primary physicians might be more effective [10].

Furthermore, the results of the PTES indicated that patient engagement in treatment was associated with factors such as age, educational background, disease knowledge, and complications. This tendency is consistent with previous findings by Baars et al. [10], who reported that younger and more highly educated patients strongly prefer SDM, and by Siegel et al. [9], who identified physician-side barriers—time, reimbursement, and tools—to implementing SDM in IBD care. Importantly, PTES should not be regarded as equivalent to validated scales such as SDM-Q-9 or CPS [11], but rather as an exploratory indicator to capture the degree of patient engagement in treatment. Owing to its simplicity, PTES may be useful in large-scale surveys and in clinical practice, for example, when pharmacists identify patients with low levels of engagement during follow-up visits and provide targeted education or support. In Xu et al. [12], a large online survey of 1,118 Chinese IBD patients found that younger age, higher educational level, and greater knowledge of shared decision-making were significantly associated with a preference to participate in SDM. In contrast, although our study also observed significant associations between these factors and SDM, no such associations were found using the Patient Treatment Engagement Scale (PTES). This divergence may be due to the different constructs measured by SDM vs. PTES—SDM concerns involvement in decision-making processes, whereas PTES probes motivation and engagement in the implementation of treatment, which may depend on different or additional influencing factors.

Moving forward, PTES requires further validation, including correlation testing with SDM-Q-9 and CPS, as well as assessment of internal consistency and test–retest reliability, to establish it as a reliable tool in clinical practice.

This study has several limitations. The cross-sectional design makes it difficult to establish causal relationships. There is potential for sampling bias due to the internet-based survey and response bias in the self-reported measurement of SDM engagement. Additionally, only a limited number of factors were examined, potentially overlooking other important influencing factors.

Moreover, given these methodological limitations, the generalization of our findings to the overall clinical population of IBD patients should be made with caution.

Future research should address these limitations by employing longitudinal designs, using more diverse sampling methods, and incorporating objective measures of SDM engagement. Further exploration of disease-specific factors influencing SDM in IBD could provide more targeted insights for improving patient care and outcomes.

Furthermore, both our study and that of Matsuoka et al [13] highlight the pivotal role of “trust in the treating physician” as a determinant of patient decision-making and satisfaction. However, while Matsuoka et al. focused solely on ulcerative colitis (UC) and emphasized the quality and content of information provided by physicians, our study included both UC and Crohn’s disease (CD) and comprehensively analyzed background factors such as the number of treatment options, the presence of complications, the number of trusted healthcare professionals, and the availability of supportive communities. Importantly, our findings suggest that for CD patients, multidisciplinary collaboration and supportive communities play an even more significant role, underscoring the need for disease-specific approaches to patient support.

Nevertheless, recent web-based studies support the feasibility and interpretability of internet surveys in IBD. In Japan, Hibi et al. [14] reported that bowel urgency and related accidents were among the most impactful symptoms in the Japanese CONFIDE cohort, highlighting communication gaps between patients and HCPs. A global analysis further confirmed broad impacts across the US, Europe, and Japan [15].

## Conclusion

This study examined patient treatment engagement in inflammatory bowel disease (IBD) using the Patient Treatment Engagement Scale (PTES). The results demonstrated that multiple factors—including the number of treatment options, complications, trust in the treating physician, and the number of trusted healthcare professionals—were associated with patients’ engagement in treatment. Notably, consistent with prior SDM research, trust in the primary physician emerged as a pivotal determinant of engagement, reinforcing the importance of the doctor–patient relationship.

Disease-specific differences were also observed. For ulcerative colitis patients, treatment options and trust in the physician were particularly influential, while for Crohn’s disease patients, the number of trusted healthcare professionals and the presence of supportive communities played a more prominent role, reflecting the complexity of CD management.

These findings suggest that PTES may serve as a practical and simplified tool to capture patient engagement in clinical settings, while also highlighting the need for tailored strategies: strengthening physician–patient trust and presenting clear treatment options for UC, and enhancing multidisciplinary collaboration and social support for CD.

## Supplementary Information

Supplementary Material 1.

## Data Availability

No datasets were generated or analysed during the current study.

## Abbreviations

SDM: Shared Decision Making
PTES: Patient Treatment Engagement Scale
IBD: Inflammatory Bowel Disease
UC: Ulcerative Colitis
CD: Crohn's Disease
QOL: Quality of Life
MHC-SF-J: Mental Health Continuum Short Form Japanese version
FDR: False Discovery Rate
VIF: Variance Inflation Factor
τ: Kendall's Tau
β: Standardized Beta Coefficient
