# Supplementary material for: Patient treatment engagement scale (PTES) for inflammatory bowel disease: exploring background factors
Source: BMC Res Notes. 2026 Sep 21;19:356. doi: 10.1186/s13104-026-07664-2 (PMC13595684; doi:10.1186/s13104-026-07664-2)
Supplement: Supplementary file 1 [file 13104_2026_7664_MOESM1_ESM.docx]

**Overall Aim of the Research**
This study aims to improve the quality of life of patients with inflammatory bowel disease by examining the influence of multiple factors, including the Mental Health Continuum Short Form-J and Shared Decision Making. The survey questionnaire is structured across five domains: well-being, medical information gathering, treatment selection, disease status, and privacy. It is designed to comprehensively evaluate various factors that reveal the extent to which patients are involved in determining their treatment plans. In our previous report, we examined the applicability of MHC-SF-J⁴⁾. In the present study, we exploratively analyze how psychosocial factors, economic factors, online medical information gathering, and disease status are involved in the shared decision-making process of IBD patients. Figure 1 shows an excerpt from the questionnaire.


**1. Well-being Survey**

These questions concern how you have been feeling during the past two weeks. Please check the box that best represents your experience or feelings. ☑︎

How often have you felt this way during the past two weeks?

|  | **Never** | **Once or twice (Once per 14 days)** | **About 2 times per two weeks (Once per 7 days)** | **About 4-6 times per two weeks (Once per 3 days)** | **Almost every day (Once per 1-2 days)** | **Every day** |
| --- | --- | --- | --- | --- | --- | --- |
| 1. I felt happy |  |  |  |  |  |  |
| 2. I felt motivated to live or passionate about life |  |  |  |  |  |  |
| 3. I experienced satisfaction or fulfillment |  |  |  |  |  |  |
| 4. I can contribute to society |  |  |  |  |  |  |
| 5. I feel I belong to some community (home, school, company, hobby group, internet, etc.) |  |  |  |  |  |  |
| 6. I thought society was becoming a more livable place for me |  |  |  |  |  |  |
| 7. I felt that people are fundamentally good |  |  |  |  |  |  |
| 8. I thought I could accept the way society functions |  |  |  |  |  |  |
| 9. I basically like my personality |  |  |  |  |  |  |
| 10. I am good at handling everyday responsibilities |  |  |  |  |  |  |
| 11. I thought I had warm and trustworthy relationships |  |  |  |  |  |  |
| 12. I had experiences that challenged me to grow and become a better person |  |  |  |  |  |  |
| 13. I thought I could express my ideas and opinions with confidence |  |  |  |  |  |  |
| 14. My life has direction or meaning |  |  |  |  |  |  |
| 15. I felt joy in daily life (additional item) |  |  |  |  |  |  |
| 16. I felt positive in daily life (additional item) |  |  |  |  |  |  |

**2. Medical Information Gathering Survey**

(1) Please indicate the number of familiar communities (home, school, company, hobby groups, internet email, social media such as LINE, FB, Twitter, etc.) with which you interact face-to-face, by phone, or other means in your current life.

Example: If you have 1 home, 1 school, 2 FB groups, 1 Twitter group, that's 5 communities, so select 4-6.

□ 13 or more　　□ 10-12　　□ 7-9　　□ 4-6　　□ 1-3　　□ 0 (None)

(2) Please indicate the number of familiar communities (home, school, company, hobby groups, internet email, social media such as LINE, FB, Twitter, etc.) with which you can discuss your illness in your current life.

Example: If you have 1 home, 1 school, 2 FB groups, 1 Twitter group, that's 5 communities, so select 5 or more.

□ 5 or more　□ 4　　□ 3　　□ 2　　□ 1　　□ 0 (None)

(3) Please indicate the number of internet information sites you trust regarding your current treatment. □ 5 or more　□ 4　　□ 3　　□ 2　　□ 1　　□ 0 (None)

(4) Please indicate how you use internet information sites regarding your current treatment. (Multiple selections allowed) □ Viewing information □ Asking questions to information providers □ Writing/posting your own information □ Not using

**3. Treatment Selection Survey**

(1) Please indicate the degree to which you were involved in your physician's decision-making regarding your treatment plan (most significant event such as surgery, etc.).

□ Involved　　□ Somewhat involved　　□ Neither　　 □ Barely involved　□ Not involved

(2) Please indicate the number of treatment options (including alternatives) that your physician presented when determining your treatment plan (most significant event such as surgery, etc.).

Example: If surgery was selected, and other treatment options included 2 types of drug therapies, nutritional therapy, and leukocytapheresis, that's 5 options, so select 5 or more.

□ 5 or more　□ 4　　□ 3　　□ 2　　□ 1　　□ 0 (None)

(3) Please indicate which medical staff you trust regarding your treatment. (Multiple selections allowed) □ Primary physician　□ Other physicians　□ Nurses　□ Hospital pharmacists　□ Pharmacy pharmacists □ Nutritionists　□ Clinical laboratory technicians　□ Physical therapists　□ Occupational therapists　□ Others

**4. Please Answer About Your Illness**

(1) Condition: ① Crohn's disease　② Ulcerative colitis　③ Difficult to differentiate between Crohn's disease and ulcerative colitis　④ Others

(2) Current severity: ① Mild　② Moderate　③ Severe　④ Fulminant　⑤ Others

(3) Age: _____ years (please fill in)

(4) Gender: Male　Female　Other

(5) Years since diagnosis: _____ years (please fill in) 　 (6) Current disease phase: ① Remission　② Active　③ Unknown (not informed)　④ Others

**5. About Your Privacy (Please provide information to the extent possible; not mandatory)**

(1) Recipient certificate for specified medical expenses (designated intractable diseases):　Yes/No

(2) Your annual income (0 yen if none): ______ million yen (please fill in)

(3) Household annual income (0 yen if none): ______ million yen (please fill in)

(4) Your employment status: Full-time employee・Part-time・Temporary worker・Contract employee・Student・Self-employed・Unemployed・Others

(5) Highest education level (including current enrollment) ① Junior high school・② High school・③ Technical college/Vocational school/Junior college ④ University ⑤ Graduate school (Master's/first-term doctoral program) ⑥ Graduate school (Doctoral program)

(6) Spouse: None・Yes

(7) Family members living together (excluding yourself): _____ people (please fill in)

(8) Prefecture of residence: _____
